# Genetic Variations on Chromosome 6p21 Are Associated with Asthma Risk and Disease Severity: A Case–Control Study from Pakistan

**DOI:** 10.3390/genes15121608

**Published:** 2024-12-17

**Authors:** Aqsa Aslam, Susanne J. H. Vijverberg, Anke-Hilse Maitland-van der Zee, Muhammad Farooq Sabar

**Affiliations:** 1 Center for Applied Molecular Biology (CAMB), University of the Punjab, Lahore 54590, Pakistan; aqsaaslam14@gmail.com; 2 Pulmonary Medicine, Amsterdam UMC, Location AMC, University of Amsterdam, Meibergdreef 9, 1105 AZ Amsterdam, The Netherlands; a.h.maitland@amsterdamumc.nl; 3 School of Biochemistry and Biotechnology, University of the Punjab, Lahore 54590, Pakistan

**Keywords:** asthma severity, genetic association, case-control study, genomic variants, low- and middle-income countries

## Abstract

Background: Genetic factors play a role in asthma severity. However, low- and middle-income countries have minimal contribution to genomic asthma research. The current study investigates the influence of an important genetic asthma region (6p21) on severe asthma in a cohort of asthmatics in Pakistan. Materials and Methods: In this case–control study, mild to severe asthmatic patients (*n* = 255) and controls (*n* = 260) were enrolled from Lahore, Pakistan. Blood samples were collected, and genomic DNA was extracted for the genotyping of 11 single nucleotide polymorphisms located in the 6p21 region. Severe asthma was defined based on the defined daily dose of inhaled corticosteroids equivalent to 200 mcg of beclomethasone dipropionate (as per the global initiative for asthma guidelines). An additive genetic model was followed to find the associations between these variants and the outcome. Univariate and multivariate logistic regression, adjusted for confounders, was performed. Odds ratio (OR), 95% confidence interval (95% CI), *p*-value, and q-values after FDR adjustment were estimated. Results: The genetic variants rs3025028, rs987870, and rs3025039 showed strong associations with the incidence of asthma with odds ratios of 1.58, 1.62, and 2.70 (95% CI = 1.16–2.16, 1.15–2.30, and 1.40–5.39, respectively). Further stratification analysis to study the risk of severe asthma also revealed markedly significant associations for rs3025020 and rs1799964 (OR = 2.28 and 2.99; 95% CI = 1.39–3.86 and 1.75–5.33, respectively). However, the SNPs rs2070600, rs987870, and rs3025039 also showed a significant relationship with the severity (OR = 2.34, 1.75, and 2.72; 95% CI = 1.02–5.97, 1.07–2.98, and 1.11–7.71, respectively), but FDR-adjusted q-values were insignificant (0.10, 0.07, and 0.07, respectively). Conclusions: The 6p21 region variants rs3025028, rs987870, and rs3025039 are associated with the incidence, whereas rs3025020 and rs1799964 are associated with the risk of more severe asthma in the Pakistani population.

## 1. Introduction

Asthma is a heterogeneous, non-communicable disease characterized by episodes of breathlessness, chest tightness, cough, wheezing, bronchial hyperresponsiveness (BHR), and reversible airflow obstruction. These recurrent and unpredictable exacerbations can lead to severe hospitalizations and fatal outcomes [1]. According to the Global Burden of Disease estimate, it affected approximately 262 million individuals globally in 2019, resulting in 461,000 deaths, equating to about 1000 deaths per day [2]. Asthma disproportionately affects low- and middle-income countries (LMICs) [3], including Pakistan, where 6–9% of urban youth suffer from severe asthma [4]. However, inadequate access to healthcare, insufficient medical infrastructure, and a lack of awareness and education hamper the effective management of asthma in LMICs. Furthermore, the diverse environmental and epigenetic landscape also contributes significantly to amplifying the disease condition [5]. Traffic-related air pollution has been associated with bronchial asthma in various studies [6,7,8]. Moreover, indoor air quality and household air pollution contribute to exacerbating asthma symptoms [9,10]. Nonetheless, the genetic profiles differ across ethnic groups, and genomic studies from these populations are lacking, while results may differ between racial/ethnic groups. This is widening the gap between the available genetic pool of knowledge, necessitating more inclusive studies from low-resource, non-white populations with limited research and healthcare expenditure [11]. Building the indigenous capacity of the local researchers is pivotal to making this shift in paradigm [3].

Moreover, a diverse genomic understanding of the disease progression in underrepresented populations is necessary to comprehend the role of genetic factors in asthma incidence and severity [12], as they can bring a change in the expression or function of proteins involved in regulatory pathways and airway inflammation. They are also the key modulators of treatment response and improve asthma control [13,14].

The short arm of chromosome 6 houses several key genes in the 6p21 region that are important in autoimmune regulation and inflammatory pathways. Mounting evidence has been provided by multiple studies validating its pivotal role in respiratory diseases such as asthma and COPD [15,16,17,18]. The genetic variants of *TNF*, *LTA*, *VEGF*, *AGER*, and *HLA* genes residing in the 6p21 region are involved in the development and progression of asthma and thus are substantial in understanding its pathogenesis [19]. The current study is designed to investigate whether key genetic variants from this region are associated with the occurrence and the severity of asthma in the Pakistani population, as the diverse genetic makeup and socio-economic challenges of Pakistan present a unique case for studying asthma severity and its genetic underpinnings in an underrepresented population.

## 2. Materials and Methods

This case–control study was designed in good accordance with the Global Initiative for Asthma (GINA-2018) guidelines [20], adhering strictly to the STROBE checklist [21] and following the ethical principles outlined in the Declaration of Helsinki [22]. This study was also approved by the Ethical Review Board, University of the Punjab (Ref: D/1629/UZ). All participants signed written consent.

### 2.1. Study Population

A total of 255 asthmatic subjects and 260 healthy controls were enrolled in the study. Participants were recruited from the tertiary care hospital, Gulab Devi Chest Hospital, in Lahore, Pakistan, between October 2017 and December 2018. Owing to the urban–rural disparities in the healthcare system in Pakistan, this specialized chest hospital welcomes patients from diverse backgrounds across Pakistan seeking intensive respiratory care; thus, varied representativeness was possible. The asthmatics were included if they were ≥16 years old, had physician-diagnosed asthma, and were on asthma medication for at least the last six months. Exclusion criteria were chronic bronchitis, tuberculosis, pneumonia, emphysema, or any other chronic respiratory disease. However, the controls were randomly recruited for the absence of asthma or any other respiratory disease.

**Figure 1:**
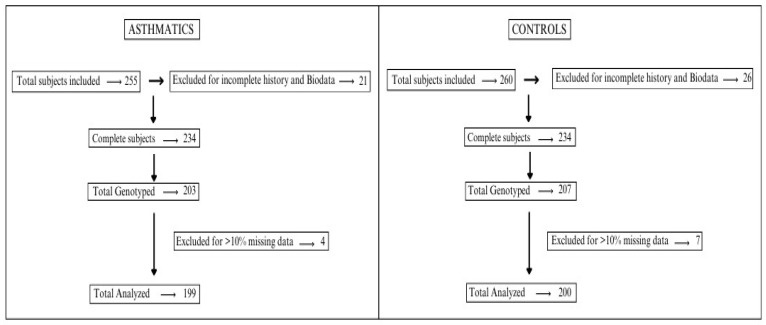
Flowchart of study participants included in this study [23].

Figure 1 outlines the number of subjects enrolled and analyzed in the study.

### 2.2. Clinical Data

Demographic and clinical data were collected on an open-ended questionnaire specifically designed for this study. An anthropometric examination was conducted of the participants, and data on treatment history and medication use were carefully recorded from hospital records and prescriptions. Proper inhalation technique was examined during recruitment, and therapy adherence was self-reported by the patients in response to the questionnaire.

### 2.3. Outcome Definition

The outcome of this study was asthma incidence and severity under the influence of genetic variants. It was defined based on the physician’s diagnosis, and the severity was assessed according to the definition in GINA (2018) guidelines: ≥1 exacerbation requiring an emergency room visit and/or ≥2 asthma attacks managed with oral corticosteroids (OCS) in the past 12 months [24]. It was further strengthened by the therapeutic pressure (treatment steps calculated based on the defined daily dose, DDD [25], of ICS administered to subjects) as per GINA-2018 [20]. The subjects were considered severely asthmatic if they were at steps 4–5 of the asthma treatment. However, those in steps 1–3 were grouped together as mild to moderate (non-severe). The asthmatics were compared against controls, and further stratification analysis was performed between severe and non-severe asthmatics.

### 2.4. Collection of Blood Samples and Genomic DNA Extraction

About 5 ml of peripheral blood was collected from the participants as per the standard operating procedure (SOP). The organic method of genomic DNA extraction using phenol, chloroform, and isoamyl alcohol (PCI) [26] was used on whole blood. The qualitative and quantitative check of DNA was performed on 1% agarose gel electrophoresis and UV/Visible spectrophotometer (Molecular Devices, CA, USA).

### 2.5. Selection and Specifications of the SNPs

Eleven SNPs (rs1800629, rs1799964, rs1041981, rs2070600, rs3025020, rs3025028, rs3025039, rs114444221, rs2395185, rs987870, and rs9273349) residing in the 6p21 region, with a potential association with the risk of severe asthma and validated in various populations, were selected to study their association in the Pakistani population. The review of the literature for the selected SNPs is detailed in Supplementary Tables S1 and S2.

**Figure 2:**
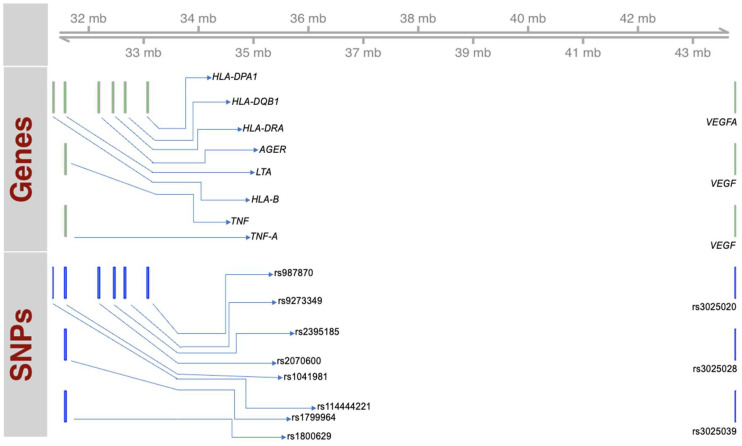
Schematic representation of the genes and SNPs located on chromosomal region 6p21, included in the study. Picture created in R using BiocManager [27].

The selection of these SNPs was based on: (1) The SNPs belong to the *tumor necrosis factor α* (*TNFa*), *lymphotoxin α* (*LTA*), *advanced glycosylation end product-specific receptor* (*AGER*), *vascular endothelial growth factor* (*VEGF*), and *human leukocyte antigen* (*HLA*) genes’ family, i.e., *HLA-B*, *HLA-DRA*, *HLA-DPA1*, and *HLA-DQB1*, and have shown a reported association with the onset and/or severity of asthma in other populations. (2) Greater than 5% minor allele frequency (MAF) has been reported for these SNPs in different populations. Supplementary Table S3 summarizes the specifications of these SNPs; and Figure 2 presents a schematic representation of these SNPs and respective genes on 6p21 region.

### 2.6. Genotyping

The SNPs were amplified through multiplex PCR reactions. However, whole genome sequencing was not feasible due to budget constraints. Therefore, we genotyped the SNPs using a cost-effective ABI Prism SNAPshot^TM^ Multiplex Kit (Applied Biosystems, Foster City, CA, USA) method. The genotypes were detected by fluorescent signals obtained from capillary electrophoresis run on the ABI PRISM 3130XL genetic analyzer (Applied Biosystems, Foster City, CA, USA) and analyzed with the GeneMapper IDX software v1.4 (Applied Biosystems, Foster City, CA, USA).

### 2.7. Genotyping Quality Control

The accuracy, integrity, and reliability of the genotype results were checked through stringent quality control (QC). The “genetics” package [28] of R [29] was used to calculate the call rate of the genotypes by setting the threshold to 95% and to identify the samples with more than 10% failed genotypes. Genotyping quality was considered poor for the samples failing at the above-mentioned criteria and, hence, were excluded.

Possible deviations from Hardy–Weinberg equilibrium (HWE) were also checked by the R (version 4.2.1) package, “HardyWeinberg” [30,31]. Allelic and genotypic frequencies were estimated, and the failed samples and/or SNPs were further removed.

### 2.8. Statistical Analysis

R version 4.2.1 [29] was employed to perform the statistical analysis. A complete case analysis approach was adopted in this study, and the samples with missing data were excluded from further analysis. The median and interquartile range (IQR) of continuous variables were reported, whereas categorical variables were represented as numbers (percentages).

The “genetics” package in R was employed to calculate the allelic and genotypic frequencies. Minor allele frequency (MAF) was determined by setting the threshold to 5% (0.05) least occurrence in the population. Risk alleles of the studied SNPs were identified from the literature, and an additive genetic model was followed for the analysis. Cleaning and coding of the data were also performed accordingly, based on the number of copies of the risk allele, i.e., “0”, “1”, and “2”. The variants were analyzed for their association with the risk of asthma incidence and severity by univariate and multivariate logistic regression among controls and asthmatics (mild to moderate and severe asthma groups), adjusted for gender and age (in years, continuous). OR, 95% CI, *p*-value, and FDR-adjusted values were recorded.

## 3. Results

The study participants with incomplete history (asthmatics *n* = 21; controls *n* = 26) were excluded from further analysis. A total of 234 true controls and 234 asthmatic participants (ages 16–90 years) were finally included. However, due to financial constraints, a limited number of subjects were randomly selected for genotyping (*n* = 203 asthmatics and *n* = 207 controls (Figure 1)). The results were further screened for their integrity by a stringent genotype quality control (QC) and 10% missingness. Hence, a total of 199 asthmatic and 200 control subjects were analyzed. The study population’s baseline characteristics are summarized in Table 1.

### 3.1. Genotyping Quality Control

All the SNPs passed the predefined quality threshold of call rate and Hardy–Weinberg equilibrium (HWE) except rs9273349, which had a call rate of 93% and *p*-value < 0.01; therefore, it was excluded. Failed asthmatics (*n* = 4) and control (*n* = 7) samples were also removed. Supplementary Table S3 details the specifications of the studied SNPs. It lists the relevant genes, alleles, position in bp (base pairs), MAF (minor allele frequency), and the type of the variant.

Furthermore, the allelic and genotypic frequencies of the studied SNPs in both asthmatics and controls are listed in the Supplementary Tables S4 and S5, respectively. The values are in good accordance with the reported frequencies across different populations.

### 3.2. Study Population

The characteristics of the study population are outlined in Table 1. 87% of participants had adult-onset asthma, and about half of the asthmatic population (49%) was male. This was a bit lower in comparison to the ratio of males in controls (56%). The median age was 40 years (IQR: 26–58) for asthmatics and 29 years (IQR: 23–40) for controls. A total of 67 participants (34%) had mild to moderate asthma, while 132 (66%) had severe asthma. Severe asthmatics were marginally overweight with a median BMI of 26 (IQR: 23–28), and half of the population had a positive family history of asthma (50%). More than half of the population (57%) had a poor adherence to therapy and a higher number of exacerbations and hospitalizations per year.

### 3.3. Genetic Predictors of Asthma Incidence and Severity

#### 3.3.1. 6p21 SNPs and Asthma Incidence

**Figure 3:**
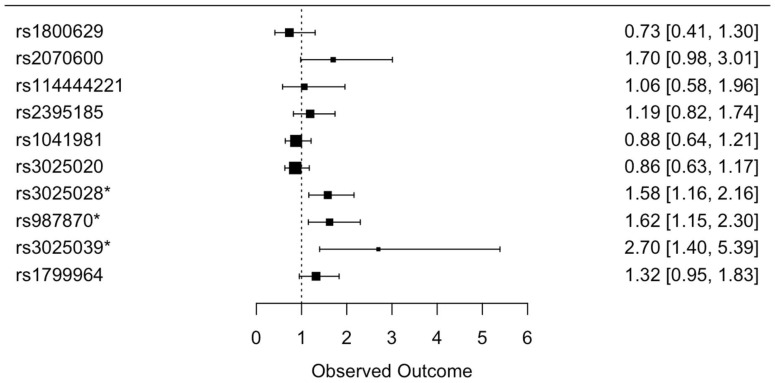
Forest plot showing the results of the multivariate logistic regression analysis for the association of genetic variants and asthma incidence. The value legends represent age- and gender-adjusted odds ratios and 95% confidence intervals (OR (95% CI)). The significantly associated risk variants are distinguished with an asterisk * [23].

A statistically significant association (after adjusting for age and gender) between rs3025028, rs987870, and rs3025039 SNPs and the risk of asthma incidence has been observed (Supplementary Table S6). However, rs2070600 does not show a statistically significant relationship with the risk of asthma with a p-value of 0.06 that turns into 0.15 after FDR adjustment (see Supplementary Table S6 and Figure 3).

#### 3.3.2. 6p21 and Asthma Severity

**Figure 4:**
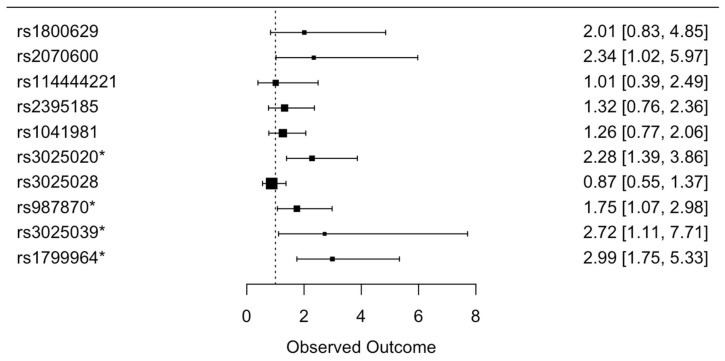
The forest plot shows multivariate logistic regression analysis results of the association of genetic variants from the 6p21 region with asthma severity. The value legends represent odds ratios and 95% confidence intervals (OR (95% CI)) adjusted for age and gender. The significantly associated risk variants are marked with an asterisk.

The association results (OR and 95% CI) for asthma severity from the univariate and multivariate logistic regression analysis between severe and non-severe asthmatics indicate that the rs2070600, rs3025020, rs987870, rs3025039, and rs1799964 SNPs are significantly associated with more severe asthma with the odds ratios of 2.34, 2.28, 1.75, 2.72, and 2.99, respectively. However, after FDR adjustment, only rs3025020 and rs1799964 remain statistically significant (q-values: 0.005 and 0.001, respectively). (see Supplementary Table S7 and Figure 4).

## 4. Discussion

This comprehensive, case–control study on severe asthma in Pakistani subjects investigated the relationship between genetic risk profile (6q21 locus) and incidence and severity of asthma. Eleven genetic variants (SNPs) from the candidate genes (*TNF*, *AGER*, *VEGF*, and *HLA*-family) were selected from the literature for their previous associations with the incidence and/or severity of asthma and global MAF of at least 5%. The genetic variants rs3025028, rs987870, and rs3025039 showed strong associations with the incidence of asthma. On the other hand, rs3025020 and rs1799964 had marked significance in terms of the risk of more severe asthma. However, the SNPs rs2070600, rs987870, and rs3025039 also showed a strong relationship with the severity but failed to reach significant FDR-adjusted q-values. The minor allele frequencies (MAFs) of the studied SNPs showed a high degree of concordance with global MAFs, demonstrating minimal deviations from expected values. This alignment strengthens the credibility of the case–control population stratification from an LMIC.

Previous investigations of the variants studied herein found similar associations with asthma development and severity, where a high prevalence of T’ alleles of *VEGF* variants, rs3025020 and rs3025039, in the asthmatic Han Chinese population [32] was observed. Relatively small changes in FEV1 were observed for the carriers of the ‘T’ allele than the ‘CC’ genotype of rs3025039, suggesting a potential impact of this allele on lung function [33]. The *VEGFA* is a key gene in asthma development that codes for *vascular endothelial growth factor* (*VEGF*) that regulates Th2-mediated inflammation through airway remodeling and blood vessel permeability [34,35]. It is overly expressed in plasma, sputum, bronchoalveolar lavage fluid, and lung tissue of acute asthma patients, and upon inhibition, it suppresses the thickness of the basement membrane and hyperplasia of goblet cells that reduces chronic inflammation [32,36], hence making them promising therapeutic targets [37].

Various genetic variants of the *HLA* (*human leukocyte antigen*) complex located in the MHC class III region have shown associations with altered susceptibility to asthma and are involved in its pathogenesis and severity [38]. An Asian asthmatic population reported significant associations for rs987870 (located on the *HLA-DP* gene) in asthma severity [39]. Genetic polymorphisms of *HLA-DQA1* and *HLA-DQB1* have also been linked to asthma susceptibility in the northern Chinese population [38,40]. *HLA* genes are key modulators of adaptive immune response, and they can affect asthma risk through distinct immunological pathways [39]. The potential relationship between *HLA* and type 2 inflammation is a hallmark of asthma pathophysiology. Previous studies have shown that variations in the *HLA* region can influence immune responses, particularly T-helper 2 (Th2)-driven pathways. Although our study primarily focused on genetic associations, the identified SNPs may contribute to type 2 inflammation by modulating antigen presentation and Th2 cytokine production [41].

The rs1799964 variant in the regulatory region of *TNF-α* (*Tumor Necrosis Factor α*) has previously been associated with severe or difficult asthma [42]. A high frequency of the ‘T’ allele has been observed in individuals affected with bronchial asthma and RSV (respiratory syncytial virus) infection [43]. However, a study of genotypic frequencies in the Han Chinese population could not find any significant difference in this SNP between controls and asthmatic subjects [44]. Nonetheless, blocking *TNF-α* has served as a successful therapeutic target for asthma patients on corticosteroid treatment, while *LTA* facilitates the attachment of *lymphotoxin* β to the cell surface by forming heterotrimers with it when released into extracellular space [45,46].

The observed association for the coding region variant, rs2070600, of the *Advanced Glycosylation End-Product Specific Receptor* (*AGER*) gene and the incidence and severity of asthma is in line with the literature citing the similar role of this SNP in lung function decline and exacerbation frequency [47,48,49]. Furthermore, it is significantly associated with the FEV1/FVC ratio and poses a risk of more severe asthma. *AGER* gene codes for the *receptor for advanced glycosylation end product* (*RAGE*) protein that maintains a balance of pro- and anti-inflammatory cytokines and modulates inflammation in asthma [50]. rs2070600 determines the serum levels of soluble *Receptor for Advanced Glycation End Products* (*sRAGE*) proteins [51] and has been associated with lung function variations [52]. The presence of the ‘T’ allele in rs2070600 results in a substitution of glycine to serine at position 82 in the *RAGE* protein, altering its functionality and contributing to asthma severity [49].

The potential biomarkers selected here were already linked to the incidence or severity of asthma across diverse populations, building the narrative on existing knowledge. The current results confirmed the relevance of these SNPs in a new demographic. These biomarkers can serve as a targeted approach for more vigilant monitoring of patients and can be explored for their role as potential therapeutic biomarkers. However, our study highlights a significant gap in asthma genetics research in the Pakistani population and underscores the need for global collaborations to foster genomic research in LMIC populations. Furthermore, it is imperative for high-income countries to address asthma disparities in LMICs as they share a great burden of non-communicable diseases and represent a major proportion of the world population. A major shift in the environmental and lifestyle changes has led to a robust increase in allergic disorders due to prolonged exposures to the pro-allergic factors. Epigenetic mechanisms mediate the effects of these environmental factors on allergy susceptibility and highlight the need for targeted treatment strategies [53].

The paucity of genomic research is growing deeper due to poor health prioritization and lack of research infrastructure (i.e., high-tech instruments and materials costing millions, inadequately trained research personnel, and resources dedicated to health improvement) in LMICs [54]. With the advancement in NGS (next-generation sequencing) in the 21st century, Western medicine is moving swiftly towards diagnostic genome sequencing and pharmacogenomics-driven treatment, whereas the populations in LMICs are devoid of these advances or reaping bare minimum benefits from these revolutionary medical practices. The disproportionate allocation of genomic resources is creating a void in the available knowledge of clinically relevant genomic variants beyond white races and limiting our ability for a unified approach to health for all by making equal therapeutic approaches available for all populations [3]. Furthermore, there are also cultural, social, and ethical implications to the acceptability of genetic research by the communities [55]. The profound disparities speak volumes about the contextual challenges in community-based asthma assessment in LMICs [4].

Furthermore, we strictly adhered to the inclusion criteria for controls and asthmatics, but a relatively small sample size may have limited the power to detect statistically significant associations. Nonetheless, the trend of an older median age among severe asthmatics, in comparison to controls, may have influenced the likelihood of including participants of a certain age. Therefore, we take it as a weakness of this study that the controls and patients had a difference in age.

The current study has limitations due to insufficient endotypic data and the absence of phenotypic characterization of both cases and controls, primarily due to genetic focus and logistical challenges. Although the controls were selected based on the absence of asthma and major respiratory or systemic disorders, they were not explicitly screened for atopic or allergic sensitization. Future studies should address these gaps by evaluating both phenotypic and endotypic markers, including allergic sensitivity and inflammatory indicators. This can enable the identification of distinct asthma endotypes and facilitate the inclusion of ‘super-normal’ controls, free from health complications, for more robust comparison.

Another limitation is the absence of respiratory function assessments in this study, which could have further strengthened the analysis of studied SNPs and their respiratory implications. More studies focusing on these aspects are warranted to explore this region further.

## Figures and Tables

**Table 1:** The baseline characteristics of the study population.

Characteristics	Controls *N* = 200	Asthmatics (Total Population) *N* = 199	Mild to Moderate Asthma Patients *N* = 67 (34%)	Severe Asthma Patients *N* = 132 (66%)
Age Median IQR (25th–75th)	29 (23–40)	40 (26–58)	30 (22–45)	47 (30–60)
Gender Male (%)	111 (56)	97 (49)	31 (46)	66 (50)
BMI Median IQR (25th–75th)	24 (21–27)	25 (23–28)	25 (22–28)	26 (23–28)
Family history of asthma Yes (%)	-	100 (50)	36 (54)	64 (49)
Age at asthma onset (years) ≤16 >16	-	26 (13) 173 (87)	8 (12) 59 (88)	18 (14) 114 (86)
Exacerbations Median (IQR)	-	2 (1–4)	1 (1–1)	3 (2–4)
Hospitalizations Median (IQR)	-	1 (0–2)	0	1 (1–2)
GINA step 1 2 3 4 5	-	13 10 44 52 80	13 10 44 0 0	0 0 0 52 80
Asthma severity Mild Moderate Severe	-	23 (12) 44 (22) 132 (66)	23 (34) 44 (66) 0	0 0 132 (100)
Therapy adherence Poor (%)	-	113 (57)	38 (57)	75 (57)
Inhaler Technique Poor (%)	-	80 (40)	27 (40)	53 (40)
Daily dose of ICS ** (mcg/day) Median (IQR)	-	500 (400–1000)	250 (200–400)	500 (500–1000)

** Equivalent of 200 mcg beclomethasone dipropionate [23].

## Data Availability

Data will be made available upon reasonable request.

## Supplementary Materials

The following supporting information can be downloaded at: https://www.mdpi.com/article/10.3390/genes15121608/s1.
