# Supplementary material for: Genetic Variations on Chromosome 6p21 Are Associated with Asthma Risk and Disease Severity: A Case–Control Study from Pakistan
Source: Genes (Basel). 2024 Dec 17;15(12):1608. doi: 10.3390/genes15121608 (PMC11675446; doi:10.3390/genes15121608)
Supplement: Supplementary file 1 [file genes-15-01608-s001.zip › genes-3335759-supplementary.pdf]

Supplementary Materials for genetic biomarkers of 6p21 region in asthma

Supplementry Tables S1 and S2 comprehensively list the SNPs and their implications in asthma based on the previous literature. Table S3 includes the SNPs which are identified and validated in candidate gene association studies while Table S4 lists the SNPs identified through GWASs. Risk allele was also identified from these studies.

Supplementary Table S1. Review of the literature for SNP selection involved in asthma predisposition and ICS response.

Gene, SNP	Reference	Number of patients & Parameters (Candidate-gene studies)	Risk allele	Outcome of the study
TNF-a rs1800629	(Almomani *et al.* 2013)	180 Caucasian children on ICS therapy<br>With an additional LABA for >5 years and LTRA for <5 years old	G	The G allele is strongly associated with the presence of difficult and severe asthma, and individuals carrying the GG genotype are at a higher risk of experiencing difficult asthma in the pediatric population, even when treated with ICS and LABA.
TNF rs1799964			C	80.8% in difficult asthma<br>66.9% in mild to moderate.
LTA rs1041981			C	24.6% in severe asthmatics and 17.4% in

				M/M asthmatics. The difference was not significant.<br><br>62.5% in difficult asthmatics.<br>Not significantly associated.
VEGFA rs3025039	(Wan *et al*. 2019)<br><br>(Lu *et al*. 2016b)	128 mild to moderate asthmatic children of Chinese origin, treated with budesonide.<br><br>Cross-sectional case-control study involving 226 Han Chinese individuals with asthma and 245 healthy controls, the role of *VEGFA* polymorphism in asthma was assessed.	T<br><br>T	Asthmatic children who carry the T allele exhibited a smaller but significant change in FEV1 compared to CC carriers. Additionally, these T allele carriers showed changes in FEV1/FVC, indicating a potential association with the response to ICS treatment in asthmatic children.<br><br>T allele is significantly more prevalent in asthma group.

VEGFA rs3025028	(Simpson *et al.* 2012a)12/17/2024 4:31:00 PM	Longitudinal birth cohort study of school children (5 years = 660, 8 years = 638 in one cohort), followed by cross sectional replication in adults (n = 596) from Manchester, and Croatian children (n = 403).	C	C allele homozygotes had significantly higher lung function in school going children with asthma.<br>Serum *VEGF-A* was higher in CC versus GG homozygotes.<br>It is significantly associated with airway function parameters from childhood through adulthood.
VEGFA rs3025020	(Lu *et al.* 2016b)	Cross sectional case control study of 226 Han Chinese asthmatics and 245 healthy controls assessing the role of *VEGFA* polymorphism in asthma.	T	T allele is significantly more prevalent in asthma group.

Supplementary Table S2. Genetic variants identified by GWAS and included in the analysis.

Gene, SNP	Reference	Number of patients & parameters (GWAS)	Risk allele	Outcome of the study
HLA-DRA rs2395185	(Li *et al.* 2012)	813 cases and 1564 controls	T	T allele is associated with an increased risk for asthma.<br>TT is the potent genotype for elevated risk.

AGER rs2070600	(Repapi *et al.* 2010a)	20,288 individuals of European ancestry	T	GWAS for the assessment of lung function.<br>High expression in lungs is associated with higher lung function, FEV1, and FEV1/FVC.
HLA-B rs114444221	(Mar *et al.* 2019)	53,031 individuals from UK biobank study	A	SNP first discovered in UK biobank study and replicated in 23andMe as well as meta-analysis.<br>A allele is associated with higher risk of childhood onset asthma and it is already linked to an increased risk of allergic disease
HLA-DPA1 rs987870	(Noguchi *et al.* 2011a)	938 Japanese pediatric asthma patients and 2376 controls.<br>Associated SNPs further replicated in Japanese (cases = 818 and controls =1032) and Korean samples (cases = 835 controls = 421)	C	It consistently demonstrated an association with the risk of pediatric asthma across three independent populations.
HLA-DQB1 rs9273349	(Moffatt *et al.* 2010)	10,365 physician diagnosed asthma patients, and 16,110 healthy controls were genotyped.	C	The C allele was found to be more prevalent in asthmatic subjects and was associated with a higher risk of asthma.

Supplementary Table S3 details the SNP specifications studied herein. It lists the relevant genes, alleles, position in base pairs (bp), minor allele frequency (MAF) and the type of the variant.

Supplementary Table S3. Specifications of the SNPs selected for the study.

**Sr. No.**	**rsID**	**Gene**	**Alleles**	**Position in BP**	**Global MAF**	**Variant type**
1	rs1800629	*TNF-A*	G/A	31575254	0.09 (A)	Regulatory region variant
2	rs2070600	*AGER-PPT2*	C/T	32183666	0.07 (T)	Missense variant
3	rs114444221	*HLA-B*	G/A	31335547	0.13 (G)	Intron Variant
4	rs2395185	*HLA-DRA*	G/T	32465390	0.29 (T)	Intron variant
5	rs1041981	*LTA*	C/A.	31573007	0.39 (A)	Missense variant
6	rs3025020	*VEGFA*	C/T	43781373	0.23 (T)	Non-coding transcript exon variant
7	rs3025028	*VEGF*	C/G	43782814	0.36 (C)	Intronic, non coding transcript exon variant
8	rs987870	*HLA-DPA1/HLA-DPB1*	G/A	33075103	0.23 (G)	Intron variant
9	rs3025039	*VEGF*	C/T	43784799	0.13 (T)	3' UTR variant
10	rs1799964	*TNF*	C/T	31574531	0.22 (C)	Regulatory region variant
11	rs9273349	*HLA-DQB1*	T/C/G	32658092	<0.01 (HPM)	TF binding site

Furthermore, the allelic and genotypic frequencies of the studied SNPs in both asthmatics and controls are detailed in the supplementary tables 4 and 5, respectively.

Supplementary Table S4. Allelic and genotypic frequencies of the studied SNPs in asthmatic subjects.

SNP	Allele frequency		Genotype frequency		
	A1	A2	G1	G2	G3
rs1800629	G = 0.931 (378)	A = 0.069 (28)	G/G = 0.862 (175)	G/A = 0.138 (28)	-
rs2070600	C = 0.899 (356)	T = 0.101 (40)	C/C = 0.803 (159)	C/T = 0.192 (38)	T/T = 0.005 (1)
rs114444221	A = 0.945 (378)	G = 0.055 (22)	A/A = 0.895 (179)	A/G = 0.100 (20)	G/G = 0.005 (1)
rs2395185	G = 0.808 (325)	T = 0.192 (77)	G/G = 0.652 (131)	G/T = 0.313 (63)	T/T = 0.035 (7)
rs1041981	C = 0.713 (288)	A = 0.287 (116)	C/C = 0.500 (101)	C/A = 0.426 (86)	A/A = 0.074 (15)
rs3025020	C =0.660 (268)	T = 0.340 (138)	C/C = 0.443 (90)	C/T = 0.434 (88)	T/ T= 0.123 (25)
rs3025028	G = 0.515 (206)	C = 0.485 (194)	G/G = 0.250 (50)	C/G = 0.530 (106)	C/C = 0.220 (44)
rs987870	A = 0.733 (289)	G = 0.266 (105)	A/A = 0.558 (110)	G/A = 0.350 (69)	G/G = 0.091 (18)
rs3025039	C = 0.906 (357)	T = 0.093 (37)	C/C = 0.812 (160)	C/T = 0.187 (37)	-
rs1799964	T = 0.701 (282)	C = 0.299 (120)	T/T = 0.493 (99)	C/T = 0.418 (84)	C/C = 0.090 (18)

Supplementary Table S5. Allele and genotype frequencies of the selected SNPs in controls.

SNP	Allele frequency		Genotype frequency			Global MAF
	A1	A2 (MAF)	G1	G2	G3	
rs1800629	G = 0.931 (380)	A = 0.069 (28)	G/G = 0.863 (176)	G/A = 0.137 (28)	-	0.09
rs2070600	C = 0.938 (375)	T = 0.062 (25)	C/C = 0.880 (176)	C/T = 0.115 (23)	T/T = 0.005 (1)	0.07
rs114444221	A = 0.931 (378)	G = 0.069 (28)	A/A = 0.867 (176)	A/G = 0.128 (26)	G/G = 0.005 (1)	0.13
rs2395185	G = 0.826 (332)	T = 0.174 (70)	G/G = 0.682 (137)	G/T = 0.289 (58)	T/T = 0.030 (6)	0.29
rs1041981	C = 0.714 (290)	A = 0.286 (116)	C/C = 0.502 (102)	C/A = 0.424 (86)	0.074 (15)	0.39
rs3025020	C =0.659 (269)	T = 0.341 (139)	C/C = 0.441 (90)	C/T = 0.436 (89)	T/ T= 0.123 (25)	0.23
rs3025028	G = 0.612 (251)	C = 0.388 (159)	G/G = 0.356 (73)	C/G = 0.512 (105)	C/C = 0.132 (27)	0.36
rs987870	A = 0.801 (314)	G = 0.204 (80)	A/A = 0.644 (127)	G/A = 0.304 (60)	G/G = 0.050 (10)	0.23
rs3025039	C = 0.961 (377)	T = 0.038 (15)	C/C = 0.923 (181)	C/T = 0.076 (15)	-	0.13
rs1799964	T = 0.742 (288)	C = 0.258 (100)	T/T = 0.552 (107)	C/T = 0.381 (74)	C/C = 0.090 (18)	0.22

Supplementary Table S6. The results of the association analysis of the genetic variants with the risk of asthma incidence between controls and asthmatics†

rsID	Crude OR (95% CI)	Adjusted** OR (95% CI)	p-value	FDR adjusted q-value
rs1800629	0.78 (0.45 - 1.35)	0.73 (0.41 - 1.30)	0.29	0.43
rs2070600*	1.69 (1.00 - 2.90)	1.70 (0.98 - 3.01)	0.06	0.15
rs114444221	1.12 (0.63 - 2.04)	1.06 (0.58 - 1.96)	0.84	0.84
rs2395185	1.15 (0.80 - 1.65)	1.19 (0.82 - 1.74)	0.34	0.43
rs1041981	0.80 (0.59 - 1.09)	0.88 (0.64 - 1.21)	0.44	0.48
rs3025020	0.91 (0.67 - 1.22)	0.86 (0.63 - 1.17)	0.35	0.43
**rs3025028**	**1.51 (1.13 - 2.04)**	**1.58 (1.16 - 2.16)**	**0.003**	**0.01**
**rs987870**	**1.39 (1.01 - 1.92)**	**1.62 (1.15 - 2.30)**	**0.005**	**0.01**
**rs3025039**	**2.71 (1.45 - 5.28)**	**2.70 (1.40 - 5.39)**	**0.003**	**0.01**
rs1799964	1.22 (0.89 - 1.68)	1.32 (0.95 - 1.83)	0.09	0.19

*Marginally significant.
**OR adjusted for gender and age.

† This work is part of the PhD thesis of the author, Ms. Aqsa Aslam;
Aslam A. 2022. A comparative study of the association of genomic variants in candidate genes of 6p21 with the pathogenicity of asthma. Ph.D. Thesis, Lahore: University of the Punjab.

Supplementary Table S7. The association analysis of genetic variants and asthma severity estimated by risk allele based additive genetic model and logistic regression between non-severe and severe asthmatics[†]

**rsID**	**Crude OR (95% CI)**	**Adjusted* OR (95% CI)**	**p-value**	**FDR adjusted q-value**
rs1800629	1.86 (0.81 - 4.19)	2.01 (0.83 - 4.85)	0.11	0.18
**rs2070600**	**2.19 (1.00 - 5.35)**	**2.34 (1.02 - 5.97)**	**0.05**	**0.10**
rs114444221	1.15 (0.45 - 2.74)	1.01 (0.39 - 2.49)	0.97	0.97
rs2395185	1.24 (0.73 - 2.18)	1.32 (0.76 - 2.36)	0.33	0.42
rs1041981	1.19 (0.74 - 1.89)	1.26 (0.77 - 2.06)	0.34	0.42
**rs3025020**	**2.01 (1.27 - 3.29)**	**2.28 (1.39 - 3.86)**	**0.001**	**0.005**
rs3025028	0.85 (0.55 - 1.30)	0.87 (0.55 - 1.37)	0.56	0.62
**rs987870**	**1.72 (1.06 - 2.88)**	**1.75 (1.07 - 2.98)**	**0.03**	**0.07**
**rs3025039**	**3.00 (1.25 - 8.36)**	**2.72 (1.11 - 7.71)**	**0.03**	**0.07**
**rs1799964**	**2.85 (1.69 - 5.00)**	**2.99 (1.75 - 5.33)**	**0.0001**	**0.001**

*Odds ratio adjusted for age and gender.

[†]This work is part of the PhD thesis of the author, Ms. Aqsa Aslam;
Aslam A. 2022. A comparative study of the association of genomic variants in candidate genes of 6p21 with the pathogenicity of asthma. Ph.D. Thesis, Lahore: University of the Punjab.
